# CRISPR-Cas technology opens a new era for the creation of novel maize germplasms

**DOI:** 10.3389/fpls.2022.1049803

**Published:** 2022-12-16

**Authors:** Youhua Wang, Qiaoling Tang, Li Pu, Haiwen Zhang, Xinhai Li

**Affiliations:** ^1^ Biotechnology Research Institute, Chinese Academy of Agricultural Sciences, Beijing, China; ^2^ Institute of Crop Sciences/National Key Facility for Crop Gene Resources and Genetic Improvement, Chinese Academy of Agricultural Sciences, Beijing, China

**Keywords:** CRISPR-Cas technology, gene editing, maize, gene function, germplasms, variety improvement

## Abstract

Maize (*Zea mays*) is one of the most important food crops in the world with the greatest global production, and contributes to satiating the demands for human food, animal feed, and biofuels. With population growth and deteriorating environment, efficient and innovative breeding strategies to develop maize varieties with high yield and stress resistance are urgently needed to augment global food security and sustainable agriculture. CRISPR-Cas-mediated genome-editing technology (clustered regularly interspaced short palindromic repeats (CRISPR)-Cas (CRISPR-associated)) has emerged as an effective and powerful tool for plant science and crop improvement, and is likely to accelerate crop breeding in ways dissimilar to crossbreeding and transgenic technologies. In this review, we summarize the current applications and prospects of CRISPR-Cas technology in maize gene-function studies and the generation of new germplasm for increased yield, specialty corns, plant architecture, stress response, haploid induction, and male sterility. Optimization of gene editing and genetic transformation systems for maize is also briefly reviewed. Lastly, the challenges and new opportunities that arise with the use of the CRISPR-Cas technology for maize genetic improvement are discussed.

## Introduction

Increasing population, climate change, and environmental stresses are crucial issues threatening global food security. It is estimated that, at present, the increase in yields of the four key global crops of maize, rice, wheat, and soybean is far below the rate of 2.4% per year, which will be needed continuously up until 2050 in order to feed the ever-growing population (Ray et al., 2013).

Maize (*Zea mays*), an important crop with the greatest global production, can contribute to satiating the demands for human food, animal feed, and biofuels (Chavez-Arias et al., 2021). However, maize grain yield is a complex quantitative trait determined by multiple genetic and environmental factors (Lu et al., 2020; Zhang et al., 2020; Chavez-Arias et al., 2021; Malenica et al., 2021; Prasanna et al., 2021). It is estimated that the yield lost *via* drought stress is currently over 20% of the maize area per year (Boyer et al., 2013; Malenica et al., 2021), and *via* high temperatures, an average of 7.4% is lost for every 1°C increase (Lobell et al., 2013; Zhao et al., 2017). Moreover, lepidopteran pests and fungal diseases can also cause over 20% or 30% of yield loss, respectively (Liu et al., 2016). Hence, the breeding of new elite crop varieties for high yield, disease resistance and abiotic stress tolerance is essential to meet the demands for maize.

Conventional plant breeding methods based on crossbreeding have been restricted in their ability to develop elite yield varieties due to the decline in natural genetic diversity, rare germplasm resources, time-consuming, intensive labor and slow breeding process (Xiao et al., 2017; Hua et al., 2019). Genetic modification (GM) technologies, such as transgenic technologies, genome editing (GE), and molecular-assisted breeding, can efficiently overcome some problems with conventional breeding, and have shown great potential for breeding elite crops with high yields under environmental stress. GM crops have been widely planted worldwide and brought multiple benefits by increasing global grain yield and quality (Abdelrahman et al., 2018; Paul et al., 2018; Montagu, 2019; Fernie and Sonnewald, 2021). In 2015, 53.6 Mha of GM maize was cultivated, representing about 1/3 of planted maize globally. In 2020, 79% of maize in the USA was GM (Pellegrino et al., 2018; Montagu, 2019; Malenica et al., 2021). However, the transgenic technologies are constrained by biological processes such as the rate of recombination and the gene-centric nature of transgenic traits.

Recently, GE technologies including ZFN, TALEN, and CRISPR-Cas, have brought unparalleled opportunities for crop breeding by precisely manipulating genetic information on a genomic scale (Hamdan et al., 2022). In particular, the CRISPR-Cas9 system and its derivative systems such as CRISPR/Cpf1, base editing (BE) and prime editing (PE) provide powerful tools to edit the plant genome by incorporating random mutations, editing multiple loci, and inducting heritable inversions and translocations (Khan et al., 2019; Capdeville et al., 2021; Fiaz et al., 2021). Moreover, developed CRISPR-dCas9 system showed an increasingly important role in the process of gene activation and repression, epigenome editing, modulation of chromatin topology, live-cell chromatin imaging and DNA-free genetic modification. Particularly, this system enables the simultaneous activation of multiple key genes that positively control different agronomic traits, which is conducive to the rapid realization of crop genetic improvement by multigene pyramiding (Zhang et al., 2019). Owing to its high efficiency, simple operation and low cost, the CRISPR-Cas technology has rapidly shown promising potential in plant functional genomics studies and the genetic improvement of crop such as rice, wheat, soybean, maize, and potato (Rao et al., 2022). This review focuses on the application and prospects of CRISPR-Cas technology in the basic science and the creation of new germplasms in maize.

## Functional annotation of maize genes using CRISPR-Cas technology

**Figure 1:**
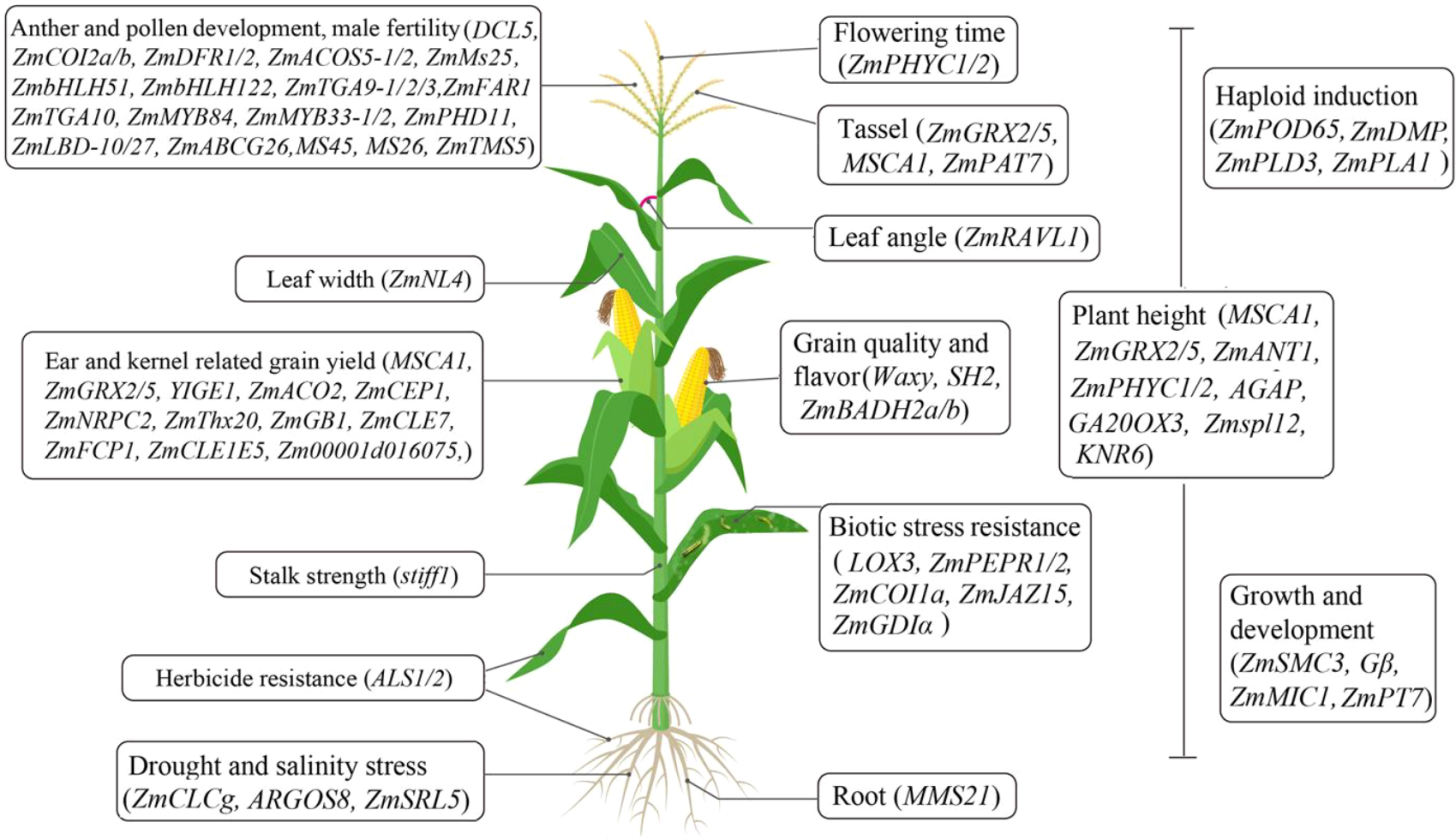
A schematic of the CRISPR-Cas technology used for the functional genomics study and generation of new germplasms in maize.

Gene-editing technology has paved an efficient and predictable way to decipher the functions of key genes and develop new germplasms in maize (Feng et al., 2016; Doll et al., 2019). Recently, the potential functions of many genes involved in maize development programs and stress responses have been well dissected using CRISPR-Cas technology (**Table 1** and **Figure 1**). For the regulation of growth and development, *ZmSMC3* has been found to be essential for sister chromatid cohesion and meiotic centromere pairing, and its knockout causes slow growth and dwarfed plants (Zhang et al., 2020). MMS21 participates in root and vegetative growth, pollen germination, and seed development by maintaining maize genome activity and stability (Zhang et al., 2021). *ZmNRPC2* controls RNA polymerase III activity and the expression of multiple genes involved in kernel development, and its knockout results in significantly reduced kernel size (Zhao et al., 2020). ZmThx20 participates in the regulation of kernel size and storage protein filling in seed, and its mutation resulted in shrunken kernel (Li et al., 2021). KNR6 and AGAP are required for vesicle trafficking, and their mutations lead to severe defects in inflorescences and roots, short ears with fewer kernels, and dwarfed plants (Li et al., 2021). CC-type glutaredoxins such as MSCA1, ZmGRX2 and ZmGRX5 mediate redox status of target proteins, and the triple knockout mutants show severely suppressed ear and tassel growth and dwarfed plant (Yang et al., 2021). YIGE1 regulates inflorescence meristem size and ear length by tuning sugar and auxin signal pathways (Luo et al., 2021). *ZmMIC1* participates in the growth of seedlings by affecting 5’-methylthioadenosine salvage and nicotianamine biosynthesis (Sun et al., 2022). Moreover, ZmPT7 and ZmPAT7 are essential for inorganic phosphate acquisition and tassel branch number, respectively (Wang et al., 2020).

**Table 1:** The potential functions and utilizations of key genes annotated by CRISPR-Cas technology.

Target gene	Potential function	Editing strategy	Phenotype	Citation
*ZmCOI2a/b*	Receptor of jasmonate signal	Knockout	Defective anther, male sterility	(Qi et al., 2022)
*ZmDFR1/2*, *ZmACOS5-1/2*,	Regulating anther and pollen development	Knockout	Defective anther and pollen, male fertility	(Liu et al., 2022)
*ZmTGA9-1/2/3 ZmMs25*	Fatty acyl reductases involved in lipid metabolism	Knockout	Defective anther and pollen, male fertility	(Zhang et al., 2021)
*ZmbHLH51, ZmbHLH122, ZmTGA9-1/2/3, ZmTGA10, ZmMYB84, ZmMYB33-1/2, ZmPHD11, ZmLBD10/27*	Regulating anther and pollen development	Knockout	Male fertility	(Jiang et al., 2021)
*ZmABCG26, ZmFAR1*	Regulating lipid metabolism	Knockout	Defective anther and pollen, male fertility	(Jiang et al., 2021)
*DCL5*	Generation of 24-nt phasiRNAs	Knockout	Defective tapetal cell, male fertility	(Teng et al., 2020)
*ZmPEPR1/2*	Regulators of defense responses	Knockout	Anti-herbivore defenses	(Poretsky et al., 2020)
*ZmGDIα*	Vesicle membrane trafficking	Knockout	Disease resistance	(Liu et al., 2022)
*ZmCOI1a, ZmJAZ15*	Jasmonate signaling components		Disease resistance Disease susceptibility	(Ma et al., 2021)
*LOX3*	Lipoxygenase	Knockout	Disease resistance	(Pathi et al., 2020)
*ZmCLCg*	Chloride transport	Knockout	Reduced salt tolerance	(Luo et al., 2021)
*ARGOS8*	Negative regulator of ethylene responses	Activating expression of *ARGOS8*	Drought tolerance	(Shi et al., 2017)
*ZmSRL5*	Maintaining cuticular wax structure	Knockout	Reduced drought tolerance	(Pan et al., 2020)
*MSCA1, ZmGRX2/5*	Modifying the redox state and the activity of their target proteins	Knockout	Suppressed meristem, ear and tassel growth, reduced plant height	(Yang et al., 2021)
*YIGE1*	Regulating ear length by affecting pistillate floret number	Knockout	Decreased inflorescence meristem size and ear length	(Luo et al., 2021)
*ZmACO2*	Ethylene biosynthesis	Knockout	Enhanced ear length, kernel number, and grain yield	(Ning et al., 2021)
*ZmPHYC1/2*	Phytochrome C	Knockout	Moderate early flowering	(Li et al., 2020)
*ZmSMC3*	Participating in meiotic centromere pairing	Knockout	Loss of sister chromatid cohesion and mis-segregation of chromosome	(Zhang et al., 2020)
*ZmMIC1*	5’-methylthioadenosine salvage and nicotianamine biosynthesis	Knockout	Interveinal chlorosis	(Sun et al., 2022)
*Gβ*	Transducers of receptor signaling	Knockout	Lethality	(Wu et al., 2020)
*ZmPOD65*	Peroxidase controlling ROS balance	Knockout	Haploid induction	(Jiang et al., 2022)
*ZmPLD3*	Phospholipase D	Knockout	Haploid induction	(Li et al., 2021)
*ZmPLA1*	Phospholipase A	Knockout	Haploid induction	(Liu et al., 2017)
*ZmDMP*	DUF679 domain membrane protein	Knockout	Haploid induction	(Zhong et al., 2019)
*Zm00001d016075*	Negatively modulating kernel row number	Knockout	Increased kernel row number and grain yield	(An et al., 2022)
*ZmCEP1*	Peptide hormones	Knockout	Increased plant height, kernel size and 100-kernel weight	(Xu et al., 2021)
*ZmNRPC2*	Second-largest subunit of RNA polymerase III	Knockout	Reduced kernel size	(Zhao et al., 2020)
*ZmThx20*	GT-2 trihelix transcription factor	Knockout	Shrunken kernels	(Li et al., 2021)
*ZmNL4*	Regulating cell division	Knockout	Reduced leaf width	(Gao et al., 2021)
*ZmCLE7, ZmFCP1, ZmCLE1E5*	CLE peptide ligands	Making weak promoter alleles	Increased multiple grain-yield-related traits	(Liu et al., 2021)
*ZmPAT7*	S-acyltransferase	Knockout	Increased tassel branch number	(Guan et al., 2022)
*ZmPT7*	Phosphate transporter	Knockout	Reduced phosphate acquisition and transport	(Wang et al., 2020)
*ZmRAVL1*	B3-domain transcription factor	Knockout	Upright plant architecture	(Tian et al., 2019)
*ZmANT1*	AP2 transcription factor	Knockout	Reduced growth rate and grain yield	(Liu et al., 2020)
*GA20OX3*	GA biosynthesis	Knockout	Semidwarf plant	(Zhang et al., 2020)
*Zmspl12*	SPL transcription factor	Knockout	Increased plant height and ear height	(Zhao et al., 2022)
*MS45*, *MS26*	Strictosidine synthase-like enzyme Cytochrome P450-like	Knockout	Male fertility	(Svitashev et al., 2015)
*AGAP*	Arf GTPase-activating protein	Knockout	Dwarfed plant, smaller ear, and small leaf	(Li et al., 2021)
*MMS21*	SUMO ligase	Knockout	Seed lethality, short root, abnormal seed/vegetative development	(Zhang et al., 2021)
*stiff1*	F-box domain protein	Knockout	Stronger stalk strength	(Zhang et al., 2020)
*ZmTMS5*	Thermosensitive genic male-sterile	Knockout	Thermosensitive male fertility	(Li et al., 2017)
*ALS1/2*	Acetolactate synthase	Knockout	Herbicide resistance	(Svitashev et al., 2015)
*ZmBADH2a/b*	2-acetyl-1-pyrroline biosynthesis	Knockout	Aromatic corn	(Wang et al., 2021)
*Waxy*	Granule bound starch synthase	Knockout	Waxy corn	(Gao et al., 2020)
*Wx*, *SH2*	Granule bound starch synthase ADP-glucose pyrophosphorylase	Knockout	Supersweet and waxy corn	(Dong et al., 2019)

For stress response, ZmCLCg positively regulates chloride transport and sodium chloride stress in maize (Luo et al., 2021). ZmSRL5 is essential for maintaining cuticular wax structure and drought tolerance in maize (Pan et al., 2020). Moreover, ZmPEPR1 restricts *S. exigua* larval growth through regulating ZmPep3-activated foliar anti-herbivore defenses (Poretsky et al., 2020). Maize G protein β subunit (*Gβ*) regulates the trade-off between growth and defense response by tuning meristem size and autoimmunity (Wu et al., 2020). Additionally, other key genes have been characterized and utilized in the development of new germplasms as follows.

## Generation of new maize germplasm resources using CRISPR-Cas technology

The CRISPR-Cas technology can overcome the limitations of conventional breeding due to the lack of available genetic resources and negative genetic linkage drag, and enables researchers to quickly and precisely modify target genes related to various traits in specific varieties, which has shown unique advantages in accelerating breeding process by generating new germplasms with more flexibility (Dong et al., 2019). Recently, CRISPR-Cas technology has been widely used to improve a variety of agronomic traits in different crops including rice, maize, wheat, barley and tomato (Liu et al., 2022). In maize, a series of new germplasms have been generated using the CRISPR-Cas technology. We with increased yield, improved quality and enhanced stress resistance, as well as male sterile lines, haploid inducers, and specialty corns We summarized them one by one in terms of improved yield, quality and special corns, haploid inducers, male sterile lines, and stress resistance.

## Yield-related traits

Maize yield has been found to be closely associated with multiple yield-related traits including ear diameter, ear length, ear row number, ear weight, 100-kernel weight, and kernel number per row (Lu et al., 2020; Zhang et al., 2020). Thus, the editing of key genes related to these traits can be conducive to improving maize grain yield. For example, *ZmCEP1* is involved in nitrate and sugar transport into the kernel, and its knockout can effectively enhance plant height, ear length, kernel size and 100-kernel weight (Xu et al., 2021). The editing of *ZmACO2* can significantly increase ear length, kernel number per row, ear weight, and grain yield in hybrids (Ning et al., 2021). For key genes positively controlling agronomic traits, CRISPR-Cas genome editing of their promoters is a potential way to generate new quantitative variations for breeding (Rodriguez-Leal et al., 2017). In maize, genome editing of cis-regulatory regions within the promoters of *ZmCLE7*, *ZmFCP1* and *ZmCLE1E5* can effectively increase meristem size and multiple grain-yield-related traits (Liu et al., 2021; Chen and Tian, 2021).

It has also been shown that ideal plant architecture for high-density planting can contribute to an increase in maize grain yield (Wei et al., 2018; Cao et al., 2022).Gene editing of *ZmRAVL1*, a positive regulator of leaf angle, can generate an upright leaf architecture and enhance high-density maize yields (Tian et al., 2019). The knockout of *ZmNL4* results in narrow leaves, which can be conducive to optimizing plant architecture for high-density planting (Gao et al., 2021). The double knockout mutant of *zmphyC1* and *zmphyC2* shows a moderate early-flowering phenotype under long-day conditions, providing valuable target genes to develop maize cultivars for adapting to different local environments (Li et al., 2020). Moreover, knockout of *Zm00001d016075*, a negative regulator of kernel row number, can increase the number of kernels per ear and grain yield (An et al., 2022). These studies have provided feasible strategies to improve maize yield *via* CRISPR-Cas genome editing.

## Specialty corns

At present, the market demand for specialty corns, such as sweet, waxy or baby corns is increasing, and CRISPR-Cas technology provides effective ways to create these specialty germplasms (Dong et al., 2019). For example, simultaneous editing of *ZmBADH2a* and *ZmBADH2b* can generate an aromatic corn by increasing the accumulation of 2-acetyl-1-pyrroline (Wang et al., 2021). Moreover, a supersweet and waxy maize has been created by CRISPR–Cas9 editing of *SH2* and *WX* (Dong et al., 2019). Recently, elite CRISPR–*wx* corn hybrids with higher yield have been developed by editing of a waxy allele in 12 inbred lines. Importantly, these CRISPR–*wx* corns are agronomically superior to introgressed hybrids, and are out of the scope of regulatory oversight over genetically modified organisms in the United States, Argentina, Brazil and Chile (Gao et al., 2020), and have initiated a new age in the global commercial production of gene-edited maize. These studies have indicated that CRISPR-Cas technology is a powerful tool to precisely develop various specialty corns with more flexibility in genetic background selection.

## Haploid inducers

Doubled haploid technology can create perfectly homozygous individuals by rapidly fixing the recombinant haploid genomes on homogenous progeny, overcoming various constraints in genetic improvement and enabling rapid evaluation of phenotypic traits in maize. If a doubled haploid technology is combined with CRISPR-Cas technology, the breeders can perform faster and more precise crop breeding (Jacquier et al., 2020; Jacquier et al., 2021). In maize, the editing of *ZmPOD65*, *ZmPLD3*, *ZmPLA1* and *ZmDMP* using CRISPR-Cas technology has successfully generated haploids, providing an approach to unravel the molecular mechanisms of haploid induction and the creation of various haploid inducers (Liu et al., 2017; Zhong et al., 2019; Li et al., 2021; Jiang et al., 2022). Furthermore, using a CRISPR-Cas9 cassette, haploid-inducer mediated genome editing was established to generate genome-edited haploids in the elite lines, which can greatly accelerate maize breeding by rapidly creating pure lines with desirable traits (Wang et al., 2019).. These studies provide potential ways to clarify the molecular mechanisms of haploid induction and breeding of haploid inducers.

## Male sterility lines

Male sterility plays an important role in hybrid seed production. Using CRISPR-Cas technology, new male sterile lines have been created utilizing male fertility genes including *ZmTMS5*, *ZmMs7*, *MS8*, *Ms26* and *Ms45* (Svitashev et al., 2015; Li et al., 2017; Chen et al., 2018). Recently, a simple next-generation hybrid seed production system was created to generate a nuclear-genetic-male-sterility (GMS) line by knocking out *ZmMS26* and its MGM maintainer line simultaneously, enabling effective production of sortable hybrid seeds labeled by a red fluorescent protein (DsRED) (Qi et al., 2020). Moreover, *ZmCOI2a*/*b*, *ZmDFR1*, *ZmDFR2*, *ZmACOS5-1/2*, *DCL5*, *ZmABCG26*, *ZmFAR1*, *ZmMs25*, *ZmbHLH51*, *ZmbHLH122*, *ZmTGA9-1/2/3*, *ZmTGA10, ZmMYB33-1/2*, *ZmMYB84, ZmPHD11*, and *ZmLBD10/27* are required for the anther and pollen development, and they have been confirmed, and will be utilized, as potential target genes for the development of novel male-sterile lines using CRISPR-Cas technology (Teng et al., 2020; Zhang et al., 2021; Jiang et al., 2021; Jiang et al., 2021; Qi et al., 2022; Liu et al., 2022).

## Stress resistance

Biotic and abiotic stresses cause devastating crop yield loss worldwide. Maize resistance is a complex quantitative trait determined by multiple genes and various environmental factors, and only few identified genes and rare elite germplasm resources are available for maize resistance breeding (Yang et al., 2017). Recently, an effective way for the creation of novel germplasm resources and breeding resistant varieties has been successfully applied *via* gene editing technology. For example, the knockout of *lox3* could enhance maize durable resistance to *Ustilago maydis* (DC.) Corda by triggering reactive oxygen species bursts (Pathi et al., 2020). CRISPR-Cas9 targeted editing of *ZmGDIα* can strongly increase maize resistance against maize rough dwarf virus without agronomic penalty (Liu et al., 2022). ZmCOIa, by interacting with ZmJAZ15, antagonistically modulates maize immunity to Gibberella stalk rot (*Fusarium graminearum* Schwabe (teleomorph *Gibberella zeae*); GSR), and the knockout of *ZmCOIa* can enhance maize resistance to GSR (Ma et al., 2021).These studies provide a good indication of the potential of gene editing technology in the breeding of maize cultivars with increased resistance to fungal pathogens or insects.

Multiple abiotic stresses including drought, extreme temperature, flooding, and soil conditions seriously affect maize production. Compared with traditional breeding methods, genome editing technologies have shown considerable potential in the breeding of stress-tolerant variants (Chavez-Arias et al., 2021; Malenica et al., 2021; Prasanna et al., 2021; Chennakesavulu et al., 2021). Stalk lodging caused by various environmental stresses is a major threat to maize yield and quality, and developing maize lines with high stalk strength has become of major interest to breeders to ensure high and stable yield (Armarego-Marriott, 2020). *stiff1* is a negative regulator of maize stalk strength, its edited allele with 2 bp deletion, caused a frameshift and an early-stop translation, conferred CRISPR-edited plants with a stronger stalk, which contributes to high density planting and preventing stalk lodging (Zhang et al., 2020). Moreover, semidwarf maize plants have been generated by editing *ZmGA20ox3* using CRISPR-Cas9 technology, which might be useful in the creation of new germplasm with stronger lodging resistance and suitable for high-density planting (Zhang et al., 2020). For drought tolerance, precisely editing the promoter sequence of *ARGOS8* leads to its upregulated expression and enhances maize grain yield under drought stress (Shi et al., 2017). Thus, CRISPR-Cas technology paves the way for the generation of novel germplasm sources for breeding stress-tolerant maize.

## Challenges and prospects

### Future and industrialization prospect of CRISPR-Cas edited maize

Breeding elite maize lines with multiple traits is a time-consuming and low efficiency process. Genome editing technology has revolutionized cereal crop improvement, and will shape future agricultural practices by integrating with traditional breeding (Hisano et al., 2021; Matres et al., 2021). In particular, CRISPR-Cas technology has shown the most exciting potential for the rapid and precise development of desirable varieties with multiple agronomic traits and stress tolerance (**Figure 1**). Now, the generation of single- and multiple-gene mutagenesis by CRISPR-Cas has become a powerful tool for functional genomics studies, to generate new germplasm resources, and to accelerate high-precision breeding processes in maize (Svitashev et al., 2015; Feng et al., 2016; Zhu et al., 2016; Agarwal et al., 2018; Doll et al., 2019; Liu et al., 2020). Notably, the combination of CRISPR-Cas technology with haploid induction or male sterility will help rapidly and predictably integrate desirable agronomic traits into new elite varieties.

It should be noted that many CRISPR-Cas-manipulated genes have been reported to improve maize traits, but very few lines have been validated by field trials and only CRISPR-waxy maize hybrids have had a limited commercial launch, in the Midwestern United States (Gao et al., 2020). Many transformed plants have only been tested for their potential breeding value in model systems or glasshouses, which may result in poor availability to develop elite maize lines suitable for field planting, and they might encounter negative effects in the field (Simmons et al., 2021). Thus, field testing is essential to evaluate whether the reported gene effects can be finally utilized in elite germplasm and maize production. To date, only 1671 genes, representing about 4.4% of the maize genome, have been tested to evaluate their field performance, and only 22 gene leads have been validated (Xiao et al., 2017; Simmons et al., 2021; Shikha et al., 2021). Thus, it is urgent to accelerate field testing of existing CRISPR-Cas-edited maize materials.

In agriculture, CRISPR-Cas technology has accelerated the process of breeding new crop varieties with improved traits, and increasing CRISPR-edited crops have been on the market, but their future depends on a scientific global regulatory framework (Ahmad et al., 2021). Among numerous CRISPR-edited maize germplasms, only limited precommercial launch of CRISPR-waxy hybrids was conducted in the Midwestern USA (Gao et al., 2020). Thus, it is more important to reframe existing regulatory policies to develop more scientific and technical criteria for the commercial production of CRISPR-Cas edited crops worldwide (Zhang et al., 2020; Ahmad et al., 2021). In addition, although CRISPR has potential to bring disruptive innovations for maize breeding, gene edited maize should consider the need of practical production and market demand to breed locally adapted and preferred crop varieties.

## Improved CRISPR-Cas technologies and transformation systems for maize

Three major obstacles to the use of CRISPR technology in maize is the low gene editing frequency, low genetic transformation efficiency and few maize materials amenable for transformation. Recently, many progresses have been made in the improvement of CRISPR-Cas technology and transformation methods for maize (**Table 2**).

**Table 2:** Improved gene editing technologies and transformation methods in maize.

Technology or system	Improved advantage	Citation
ViMeBox	Rapid positive selection by visualizing fluorescence with the naked eye	(Xu et al., 2021)
SFR-assisted CRISPR/Cas9 system	Seed fluorescent fluorescence reporter assisted selection	(Yan et al., 2021)
I-E CRISPR-Cascade tool	Mediating target gene activation	(Young et al., 2019)
Inducible CRISPR-Cas9 system	High-frequency and selectable marker-free intra-genomic gene targeting	(Barone et al., 2020)
Optimized pegRNA expression system	Improved prime-editing efficiency	(Jiang et al., 2020)
CRISPR-Cas12a Ribonucleoprotein Complexes	Improved editing efficiency	(Dong et al., 2021)
CRISPR Cas12a system	Targeted chromosomal crossovers	(Kouranov et al., 2022)
CRISPR/dCas-mediated gene activation toolkit	High-specific gene activation	(Qi et al., 2022)
PhieABEs	Improved base-editing activity and expanded target range editing windows	(Tan et al., 2022)
ISU Maize CRISPR	High-frequency targeted mutagenesis	(Char et al., 2017)
Marker-free site-specific gene insertion method	High-frequency of targeted gene insertion	(Peterson et al., 2021)
Transformable fast-flowering mini-maize	FFMM line amenable for genetic transformation	(McCaw et al., 2020)
Ternary vector system united with morphogenic genes	Increasing the efficiency of CRISPR/Cas delivery transformation and	(Zhang et al., 2019)

As for optimizing CRISPR-CAS technology to improve gene editing efficiency, a type I-E CRISPR-Cascade tool for gene activation, a ViMeBox (VIsual Maize Editing toolBox) high-frequency gene targeting method, and a seed fluorescence reporter (SFR)-assisted CRISPR-Cas9 system have been established in maize, providing effective and rapid methods to generate genome-edited plants (Young et al., 2019; Barone et al., 2020; Xu et al., 2021; Yan et al., 2021). Further, a highly efficient prime-editing tool, unrestricted by the strict NG protospacer adjacent motif (PAM) sequences, has been created by optimizing pegRNA expression in maize (Jiang et al., 2020). A selectable marker-free site-specific gene insertion can efficiently increase the frequency of targeted gene insertion (Peterson et al., 2021). Recently, CRISPR-Cas technologies have successfully manipulated targeted large fragment insertion/inversion and chromosomal crossovers in maize, providing promising methods to develop new maize varieties with multiple-traits stacking by breaking linkage drag, generating genetic diversity and facilitating trait introgression (Lee and Wang, 2020; Schwartz et al., 2020; Gao et al., 2020; Kouranov et al., 2022).

Traditional transgenic technology by overexpressing specific genes is an important technology to verify gene function and developing new crop materials with the improved traits, but its wide application is limited due to its difficulty in realizing the multi-gene polymerization and the strict regulation of genetically modified crops. Recently, the developed CRISPR-based gene activation technology has offered powerful tool for the simultaneous activation of multiple genes in a way superior to traditional transgenic technology, which provides new strategy for the manipulation of crop genetic improvement using the key genes positively controlling specific agronomic traits (Ding et al., 2022). For example, a CRISPR/dCas9-based toolkit was established to precisely and effectively activate expression of specific target genes in maize (Qi et al., 2022).

Moreover, off-target effects are frequently reported and widely existed in genome editing plants. A variety of approaches including improved CRISPR-Cas systems and new methods have been developed to eliminate the potential for off-target editing (Young et al., 2019; Modrzejewski et al., 2020; Sturme et al., 2022). It has been reported that the off-target editing in CRISPR-Cas edited maize plants could be mitigated using specifically designing guide RNAs differed from other genomic locations (Young et al., 2019). Moreover, a PAM-less/free high-efficiency adenine base editor toolbox (PhieABE toolbox) can effectively prevent off-target editing in rice, which shows wide applications in plant functional genomics and crop improvement (Tan et al., 2022).

Currently, *Agrobacterium-*mediated transformation is widely used to generate gene-edited plants, but this has major constraints due to few maize inbred lines amenable for transformation, low transformation efficiency and lower editing frequency (Ayar et al., 2013; Peterson et al., 2021; Hunter, 2021). Recently, several *Agrobacterium-*mediated transformation methods have been improved for maize genome-editing. For example, an *agrobacterium*-mediated CRISPR-Cas9 platform for high-efficiency genome editing has been used in a maize Hi II line (Char et al., 2017; Lee et al., 2019). Additionally, the Fast-Flowering Mini-Maize (FFMM) line A, previously unsuitable for genetic transformation, has now been bred for transformable lines by hybridizing it with a transformable genotype high Type-II callus parent A (Hi-II A) with line A of FFMM (McCaw et al., 2020). Moreover, a ternary vector system-based platform, employing *WUS* and *Bbm* for *Agrobacterium*-mediated transformation, can effectively increase the efficiency of transformation and gene editing, and widen the range of maize lines available for transformation (Zhang et al., 2019; Julkowska, 2019). In addition, the gene editing rate of CRISPR-Cas12a using particle bombardment was increased to over 60% in an elite maize variety (Dong et al., 2021). Thus, continuous optimization of transformation methods and gene editing systems is a prerequisite for the promotion of the application of gene editing technology in maize.

## Author contributions

YW, QT, LP, HZ, and XL were contributed to the writing of this review and approved it for publication.
